# Characterization of the normal fetal circulatory system of the ductus venosus using sound complexity parameters

**DOI:** 10.1590/1414-431X2023e13018

**Published:** 2023-11-13

**Authors:** A.S.R. Souza, C.F. Carvalho, G.F.A. Souza, R.B. Moraes

**Affiliations:** 1 Centro de Atenção à Mulher, Instituto de Medicina Integral Prof. Fernando Figueira, Recife, PE, Brasil; 2 Escola de Saúde e Ciências da Vida, Universidade Católica de Pernambuco, Recife, PE, Brasil; 3 Centro de Ciências Médicas, Universidade Federal de Pernambuco, Recife, PE, Brasil

**Keywords:** Entropy, Doppler effect, Heart diseases, Ultrasonography, Fetal heart

## Abstract

The aim of this study was to characterize the normality of the fetal circulatory system through the time between ventricular systoles of the ductus venosus in the three gestational trimesters in healthy fetuses using nonlinear methods of the complexity of the signal. A prospective cohort study was conducted at the Instituto de Medicina Integral Prof. Fernando Figueira (IMIP) from December 2019 to May 2020. Pregnant women between 11 and 14 weeks, with intrauterine pregnancy and healthy fetus were included. Patients with multiple gestation, positive screening for congenital malformation, including heart disease, and under 18 years of age were excluded. Doppler velocimetry ultrasonography of the ductus venosus was performed between the 11th and 14th weeks, 20th and 24th weeks, and 28th and 32nd weeks of gestation, and then the sound signal was extracted and segmented from the videos. To compare the means between the gestational trimesters of the approximate entropy (ApEn) and Lempel-Ziv complexity (CLZ) of the time between ventricular systoles, the Friedman test was used, with a significance level of 5%. No statistically significant difference was found between the 1st, 2nd, and 3rd trimesters regarding the mean ApEn (P=0.281) and CLZ (P=0.595) of the time between ventricular systoles of the ductus venosus. Ductus venosus systolic time was not sensitive to differentiate fetal cardiovascular dynamics between gestational trimesters. This study pioneered the characterization of cardiovascular normality by nonlinear parameters of the fetal ductus venosus in all three trimesters.

## Introduction

Screening for congenital heart disease (CHD) in pregnancy allows the programming of postnatal follow-up in order to perform procedures to treat or promote well-being in the immediate postnatal period. Of all congenital malformations, CHD is the most frequent in the postnatal period and is believed to be present in 0.9% of live births, of which 20 to 30% have severe structural defects and 3 to 5% of these die in the neonatal period (1). Despite technological advances and the expansion of ultrasound imaging sections, obstetric ultrasound still has a low diagnostic value, even though it is considered the gold standard for diagnosing CHD (2,3).

Echographic screening for CHD includes measurement and evaluation of the ductus venosus (DV). In the first trimester, abnormal flow in the DV is observed in aneuploid fetuses and in those with a cardiac defect, especially if the nuchal translucency is also altered (4). Evaluation of the pulsatility index (PI) of the DV can improve the detection rate of cardiac anomalies by 70% (5). At the end of the 2nd trimester, the PI is also associated to fetuses with growth restriction and may indicate acidemia and impending fetal death (6-8).

Currently, complexity parameters have been used to characterize the activity of the cardiovascular system in adults and children (9). However, there are still few studies in the literature on the use of these parameters for the study of fetal cardiac activity. The signals emitted by the cardiovascular system show a complex behavior that, when pathological, alters the dynamic regulatory system by emitting a signal (10). To study these signals, the Lempel-Ziv complexity (CLZ) has been used and proven to be a reliable method for differentiating time series with the level of variability similar to the cardiovascular system (11). Approximate entropy (ApEn), on the other hand, uses a mathematical approach to quantify the complexity of a system, where a low value generally indicates predictability and regularity of the time series and a high value indicates unpredictability and random variation (12).

The characterization of cardiovascular system signaling applied to time series may be a potential clinical biomarker to differentiate fetal autonomic cardiac condition at different stages of life. Thus, we aimed to use linear and nonlinear methods of computational analysis to analyze the time between ventricular systoles of the DV in the three gestational trimesters, in healthy fetuses.

## Material and Methods

A prospective cohort study was conducted involving pregnant women who were seen at a reference institution for comprehensive care of women's and children's health, Instituto de Medicina Integral Prof. Fernando Figueira (IMIP), in Recife, Pernambuco, Brazil, from December 2019 to May 2020.

The present research was initiated after approval by the Ethics and Research in Human Beings Committee of IMIP, under CAAE 12873719.0.0000.5201 and opinion number 3.780.813 of 18/12/2019, and of the Universidade Católica de Pernambuco (UNICAP), under CAAE 12873719.0.3001.5206 and opinion number 3.931.232 of 24/03/2020. All participants signed the informed consent form.

Pregnant women between 11 and 14 weeks, with intrauterine pregnancy and a live fetus were included. Patients with multiple gestations, positive screening for congenital malformation, including heart disease, and under 18 years of age were excluded.

The variables studied were maternal age, race/ethnic group, gestational age in each trimester, parity, body mass index (BMI) in the first trimester, presence of comorbidities such as chronic arterial hypertension, diabetes mellitus, systemic lupus erythematosus, and antiphospholipid syndrome, and time between ventricular systoles in the DV in the three gestational trimesters by nonlinear methods of computational analysis, ApEn, and CLZ.

Doppler ultrasonography was first performed between the 11th and 14th weeks of pregnancy, then between the 20th and 24th weeks, and finally between the 28th and 32nd weeks of gestation in the DV according to the usual technique, and was videotaped (13). All examinations were performed by a single observer specializing in fetal medicine (A.S.R.S.). In the first trimester scan, the fetal heart rate (FHR) and pulsatility index (PI) in the DV were manually calculated, the fetal crown rump length (CRL), thickness of the nuchal translucency (NT), and the tricuspid valve flow were measured, and a complete evaluation of the fetal anatomy, including the heart was performed. A specific technique was used to assess the DV waveform to avoid contamination from adjacent veins: fetal quiescence, magnification of the fetal thorax and abdomen images, right ventral mid-sagittal view of the fetal trunk, small pulsed Doppler sample (0.5-1 mm), insonation angle <30°, filter set at a low frequency, and high sweep speed (2-3 cm/s) so that the waveforms are spread allowing better assessment of the a-wave (14).

In the second and third trimesters, morphological ultrasound with Doppler velocimetry of the DV was performed, the FHR was calculated, and a detailed evaluation of the fetal heart was performed. For all trimesters, the ultrasounds followed the measurement and evaluation techniques recommended by the International Society of Ultrasound in Obstetrics and Gynecology (ISUOG) (15), Fetal Medicine Foundation (FMF) (14,15), and the “As Low As Reasonably Achievable” (ALARA) principle (16). At birth, all neonates underwent a detailed physical examination by professionals specialized in neonatology and, if necessary, underwent neonatal echocardiography to rule out congenital heart disease.

From the sonographic videos recorded, the Doppler velocimetry audios were extracted and segmented to obtain the time interval between ventricular systoles of the DV, and the files were converted from (.mpg) to (.wav) using the program Covertio 2021 (Softo Ltda, Lamarca, Cyprus, https://convertio.co/pt/). After obtaining these files, the audio signals were loaded into QuB software (version 1.4.0.1000; The MLab Edition, USA, https://milesculabs.biology.missouri.edu/QuB_Downloads.html) for manual segmentation of the time components of the pulse equivalent to ventricular systole (S) and removal of artifacts, and the time between the S waves of the DV pulses was calculated.

Subsequently, the ApEn and CLZ algorithms were calculated using a sequence of 30 S-wave intervals for each patient in all trimesters using MATLAB^®^ software (Version R2017a, MathWorks, USA, https://www.physionet.org). Recently, ApEn has been consistently used in the evaluation of heart rate variations in short time series with up to 30 s and has shown consistent results. Thus, it seems to be a reliable tool in the analysis of signs of patients who are not available for a long acquisition, as in the case of prenatal exams (17). In addition, several studies have also been carried out, showing the robustness of the Lempel-Ziv algorithm in the analysis of biological series of very short duration (<30 s), including cardiac variations in neonates (18).

Considering an Sn sequence and having N samples in a time series signal, EE(1), EE(2), EE(N), two initial parameters r and m are chosen to calculate the ApEn (Sn, m, r) for a given segment. Parameter m defines the length of the substring and r defines the similarity criterion. A time series of biological signals that has length m<N and that starts at a measure i of Sn will be represented by a vector pm(i). Two vectors pm(i) and pm(j) are said to be similar if the difference between any pair of values of these vectors is less than r, i.e., if: [EE(i+k) - EE(j+k)] < r for 0 ≤ k < m.

Considering that pm contains all subsequences of length m, i.e., [pm(1), pm(2), pm(N-m+1)] in Sn, then Cim can be defined as: Cim(r) = nim(r) / N - m+1, where nim(r) is the number of subsequences in pm that are similar to pm(i). The value of Cim(r) is a fraction of subsequences of length m that resemble subsequences of the same length that start in interval i. The value of Cim(r) for each subsequence in pm is calculated and Cm(r) is obtained by averaging the values of Cim(r). This will represent the repetitive subsequences of length m present in Sn. ApEn is finally calculated as: ApEn(Sn, m, r) = ln [Cm(r) / C(m+1)(r)]. That is, ApEn corresponds to the natural logarithm of the repetitive subsequences of length m compared with the repetitive subsequences of length m+1. Therefore, the ApEn calculation is able to estimate the probability with which the subsequent intervals will differ.

ApEn (m, r, N) is a non-linear method that has been used to investigate the complexity/regularity of signals with different lengths (N), from a minimum series (m), using a tolerance (r) of 15% of the standard deviation of the series to compare the minimum series and its increasing (m+1) with the entire series (N). It has been used to assess heart rate variability in several studies in recent years. Details about the method can be found in the article by Pincus (12).

The CLZ analysis is based on the definition described by Ziv and Lempel (19) for analyzing patterns in discrete space-time series. Its values are normally between 0 and 1. This approach initially transforms the time series followed in a binary data sequence. This initial process is carried out by establishing the average of the analyzed segment. A new sequence (S) is constructed using the average of the original segment as a threshold. If the sample value is greater than the mean, it is defined as one, otherwise it is zero. Thus, S is composed of only these two binary symbols. A scan is performed on S from its first sample to the end. When a new subsequence, which was not found in the previous scanning process, is discovered, the complexity value is increased by one. Thus, the CLZ symbolizes the number of all different subsequences contained in the original sequence. In general, the normalized complexity C(n) is often used to obtain an independent measure of sequence length: C(n) = C(n) / b(n); b(n) = N / log2(N) (19).

The CLZ is a complexity measure that was initially used to analyze symbols and later adapted to evaluate the entropy of time series samples of different natures. In the evaluation of biomedical signals, it has shown to be a good substitute for the Lyapunov exponent and can be executed from the establishment of a threshold to binarize the series, and, from a minimum sequence of characters, it manages to establish how much the series of data has variations. It also has the advantage that it can be used for both very small and very large series without requiring great computational power, which makes it a good candidate for developing software to classify series with few data (19).

The statistical analysis of the data was performed using the OriginPro software (version 8.0, USA). Initially, frequency distribution tables were generated for categorical variables, then means, variances, and standard deviations were calculated and statistical tests were applied. To evaluate data distribution, the Kolmogorov-Smirnov normality test was used. The FHR and the PI of the DV data were normally distributed in the three trimesters and analyzed by analysis of variance (ANOVA) and Tukey's *post hoc* test. The data from ApEn and CLZ of time interval between ventricular systoles were not normally distributed and Friedman's nonparametric test was performed. For all steps, P<0.05 was considered significant. We obtained a graphic representation of the ApEn and CLZ and calculated the R^2^ correlation coefficient, which values between 0 and 1 indicate a positive linear correlation, and the closer to 1, the better the fit of the model considered.

## Results

In the period studied, 40 patients were eligible for the study; nine pregnant women were excluded as three had fetal malformations, one was under 18 years of age, and five did not sign the informed consent form. In total, 31 patients were evaluated and selected for this study.

The mean maternal age was 28±5.54 years. Most patients self-reported belonging to other (nonwhite and nonblack) ethnic groups (n=26; 83.8%) and had no comorbidities (n=25; 80.6%). Mean gestational ages were 12.7±0.7 weeks, 22.6±0.81 weeks, and 29.2±0.9 weeks in the 1st (n=31), 2nd (n=27), and 3rd (n=22) trimesters, respectively. The mean parity was 1±0.95 and the mean BMI was 26.8±7.0 kg/m^2^ (Table 1).

**Table 1:** Demographic and obstetrics characteristics of study participants.

Variable	
Maternal age (years) (mean±SD)	28±5.54
Ethnic group/race (n; %)	
White	3 (9.6%)
Black	2 (6.4%)
Other	26 (83.8%)
Gestational age (mean±SD)	
Parity (means±SD)	1±0.95
Body mass index (mean±SD)	26.8±7.0
Comorbidities (n; %)	
Yes*	6 (19.4%)
No	25 (80.6%)

SD: Standard deviation. *A patient may have one or more comorbidities [chronic arterial hypertension (n=5), type 2 diabetes mellitus (n=2), systemic lupus erythematous (n=1), and antiphospholipid syndrome (n=1)].

There was a significant decrease in mean FHR in the first trimester from 160.1±7.0 bpm to 145.5±8.3 bpm in the second and 143.7±7.4 bpm in the third trimester (P=0.0001). Similarly, the PI of the DV decreased from 0.95±0.2 in the first trimester to 0.55±0.11 in the second and 0.55±0.10 in the third trimesters (P=0.001) (Table 2).

**Table 2:** Fetal heart rate and pulsatility index (PI) of the ductus venosus according to the gestational period.

Variable	1st trimester(n=31)	2nd trimester(n=27)	3rd trimester(n=22)	P (ANOVA)
Fetal heart rate (mean±SD)	160.1±7.0	145.5±8.3	143.7±7.4	0.0001
PI ductus venosus (mean±SD)	0.95±0.2	0.55±0.11	0.55±0.10	0.001

PI: pulsatility index.

**Figure 1:**
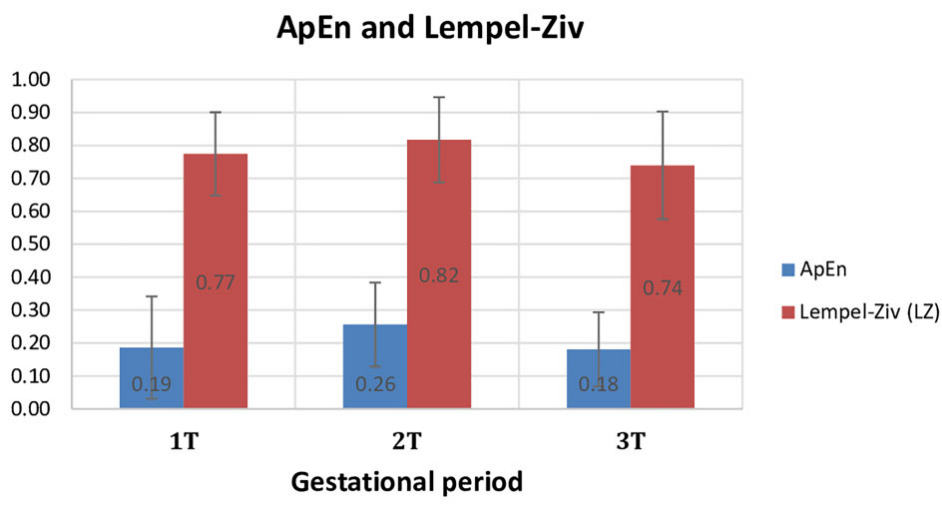
Means and standard deviations for the approximate entropy (ApEn) and Lempel-Ziv (LZ) complexity of the time interval between ventricular systoles according to the gestational trimester (T). Friedman's test, ApEn P=0.281 and LZ P=0.596.

No statistically significant difference was observed between gestational trimesters regarding mean ApEn of the time interval between ventricular DV systoles (1st trimester=0.19±0.16; 2nd trimester=0.26±0.13; 3rd trimester=0.18±0.11; P=0.281) and the mean CLZ (1st trimester=0.77±0.13; 2nd trimester=0.82±0.13; 3rd trimester=0.74±0.16; P=0.598) (Figure 1). After using a second-order polynomial trend line for the ApEn and CLZ graphs, the coefficient of determination R^2^ was 1, indicating a perfect fit.

## Discussion

In our study, no significant difference was observed in mean ApEn and CLZ of the time interval between ventricular systoles of the DV between gestational trimesters. However, we observed a decrease in FHR and PI of the DV with increasing gestational age. These results may be due to fetal cardiovascular dynamics, which is directly related to physiological determinants of cardiac function: firstly, systolic function (preload), then blood flow resistance (postload), contractile function, fetal heart rate, and finally diastolic function (20).

The DV has the function of directly conducting oxygenated blood from the umbilical vein to the heart, and its blood velocity reflects the pressure gradient between the central venous system and the umbilical vein, thus offering insight into fetal hemodynamics, proper development, and early manifestation of myocardial compromise (21).

Although it has been assumed that the DV waveform can be used to assess cardiac function, recent evidence indicates that wave speeds in particular are not related to individual cardiac performance parameters but to the entire cardiac cycle (22).

FHR and PI are two indices of linear dynamics that can be analyzed in the time and frequency domain. The variability of FHR is influenced by the autonomic nervous system, the fetal body, respiratory movements, baroreflex, and circadian processes (23). A study showed differences in FHR variability in the 1st trimester compared to the 2nd and 3rd trimesters. However, there were no differences when comparing the FHR variability of the 2nd and 3rd trimesters. These data corroborate a study conducted with chronically catheterized sheep that were exposed to normoxia in isobaric chambers without any external influences demonstrating that intrinsic influences at the end of pregnancy can affect fetal cardiac function and that the decrease in heart variability in the third trimester may reflect myocardial dysfunction in both systole and diastole (24).

On the contrary, another study found abrupt changes in fetal heart rate in the third trimester using fetal cardiotocography. However, the study was performed with fetuses in motion, which may have a direct influence on FHR accelerations (11). Another study using time series demonstrated decreased FHR variability in fetuses with growth restriction (25).

Other authors have found similar results using fetal magnetic resonance imaging, in which the slopes of heart rate variability in relation to gestational age have shown to be very similar (25). Recently, it was observed that a lower complexity and variability assessed in the third trimester may be related to absence of inflammatory conditions, imminent delivery, and fetal immunological tolerance (26).

In this study, we observed that DV waveform patterns measured by the PI are different in the first trimester compared to the second half of gestation. All the DV waveforms used in the study were of high quality and were measured manually and calculated automatically by the device. This study corroborates the literature, in that constant PI values and flow velocities were found in the DV in the second half of gestation (27).

Normally, there are no significant cardiac changes in the first trimester, whereas significant physiological changes occur in the second half of pregnancy, such as progressive increase in compliance and reduction in afterload, which would justify higher flow velocity and decreased PI of the DV at these gestational ages (28).

Some authors use the DV PI for screening for congenital heart defects in the first trimester of pregnancy. However, the shape of the Doppler velocimetric wave has more relevance, because DV wave patterns in this period expressed as velocity ratios at various inflection points are different in fetuses with heart defects (28).

The results of our study corroborated those of other authors, who suggest that direct flow during ventricular systole, end-diastole ventricular relaxation, and early ventricular diastole are directly related to advancing gestational age and do not modify the PI measurement. These reference intervals may be beneficial for assessment of fetal cardiac conditions and aid in the diagnosis of cardiovascular abnormalities (29).

ApEn and CLZ are two nonlinear dynamics indices used to differentiate cardiovascular dynamics in healthy fetuses. In the present study, these indices were used to compare time between ventricular systoles of the DV in the first, second, and third gestational trimesters. The ApEn was introduced to quantify the complexity of the system based on the theory that disease is characterized by an increased irregularity of the time series of vital signs compared to the dynamic stability of a healthy condition (30). Therefore, a low value of ApEn indicates unpredictability and random variation, and the higher the entropy value, the more complex the process (31).

The CLZ has been shown in some studies to be a reliable method to differentiate the time series of the cardiovascular system variability. It is based on the definition, for pattern analysis in discrete space-time series and its values are usually between 0 and 1 (12). This approach transforms the analyzed signal in a binary data sequence. This initial process is performed from the establishment of an average of the analyzed segment (32).

CLZ is a complexity measure that has been used to analyze electroencephalography signals in patients with Alzheimer's disease, attention deficit hyperactivity disorder, depth of anesthesia, and epilepsy among other conditions (33). It is essentially a measure of the unpredictability of a series, but it may be influenced by the set of frequencies that make up the signal (34).

In the present study, the CLZ values showed a tendency of the mean FHR to increase in the second trimester, but there was no statistical significance. The literature shows that there is a significant increase in cardiac development due to myocardiogenesis from the second trimester onward, which affects the patterns of cardiac contractility and relaxation capacity (35). The ApEn showed a lower mean in relation to the first and second trimesters, but also with no significant difference. Some studies show that in the third trimester there is less variability in the cardiac cycle dynamics, which translates into good cardiac health (20).

Another study performed with three-dimensional ultrasound using spatio-temporal image correlation observed that there is a difference in cardiovascular dynamics regarding cardiac output and ejection fraction, in that cardiac output increases with gestational age and is not different between left and right ventricles, and ejection fraction decreases with gestational age, being higher in the left ventricle (36).

The correlation coefficient R^2^ of ApEn and CLZ indicated a perfect fit of the model. Therefore, even though the statistical analysis did not show any significant difference, the two parameters showed a visible increase in the 2nd trimester and a decrease in the 3rd trimester.

The perspectives on the use of DV Doppler using linear and nonlinear methods, although limited, bring new possibilities for the understanding of fetal cardiovascular dynamics. The limitations of this study include a relatively small number of subjects studied, the capture of fetal Doppler waves that requires ideal fetal position, little fetal movement, apnea of the pregnant woman, and maternal biotype, which sometimes makes the examination extremely time-consuming. Additionally, the presence of abdominal scars can hinder the satisfactory performance of the exam and the capture of adequate images. Despite these difficulties, it is important to emphasize that the patients were followed during the entire gestational period and that there are no comparable studies in the literature. Moreover, the specialist in fetal medicine took care to capture the most adequate waves and the time required to perform the analyses. No patient was excluded after the examination and video recording.

### Conclusion

Although the use of nonlinear methods such as ApEn and CLZ are accurate and reliable techniques for the detection of changes in the cardiovascular autonomic system and fetal development, they were not sensitive enough in this study to differentiate fetal cardiovascular dynamics in systolic times (S waves) by Doppler study of the DV. However, it was possible to characterize the normal fetal cardiovascular pattern through these parameters and to observe that measures of central tendency indicated a subtle difference between trimesters.

It is possible that future studies with a larger number of women followed throughout pregnancy will find significant and sensitive markers for early detection of cardiovascular malformations. This work shows the importance of nonlinear methods for assessing fetal cardiovascular dynamics, which allow characterization of aspects that cannot be detected by classical methods of analysis.
